# An Unexpected Complete Remission of Advanced Intestinal-Type Vulvar Adenocarcinoma after Neoadjuvant Chemotherapy: A Case Report and a Literature Review

**DOI:** 10.1155/2013/427141

**Published:** 2013-11-06

**Authors:** Angela Musella, Claudia Marchetti, Laura Salerno, Laura Vertechy, Roberta Iadarola, Irene Pecorella, Pierluigi Benedetti Panici

**Affiliations:** ^1^Department of Gynecology, Obstetrics and Urological Sciences, Sapienza University of Rome, Viale del Policlinico, 155-00161 Rome, Italy; ^2^Department of Experimental Medicine and Pathology, Sapienza University of Rome, Viale Regina Elena, 324-00161 Rome, Italy

## Abstract

Vulvar cancer represents approximately 3%–5% of all gynecological malignancies. Squamous cell carcinoma is the most frequent histotype, whereas melanomas, adenocarcinomas, basal cell carcinomas, and sarcomas are much less common. Intestinal-type adenocarcinoma is a rare variant of vulvar carcinoma with only few cases found in the literature. The origin of this neoplasia is still much debated, but the most reliable hypothesis is the origin from cloacal remnants that may persist in the adult in different organs. Because of its extremely low incidence, the optimal management of this kind of vulvar cancer is still debated. We report the case of a woman affected by advanced intestinal-type vulvar adenocarcinoma, who achieved a complete clinical and pathological response after neoadjuvant chemotherapeutic treatment with platinum and paclitaxel.

## 1. Introduction

Vulvar cancer (VC) represents approximately 3%–5% of all gynecological malignancies [1]. It affects 17.3% of women aged 30–49 years, 32.1% of women aged 50–69 years, and 47.6% of women aged more than 70 years. Squamous cell carcinomas (SCC) account for 80% of cases, whereas melanomas, adenocarcinomas, basal cell carcinomas, and sarcomas are much less common. Vulvar adenocarcinomas mostly originate from Bartholin's glands [2–6], but rarely from sweat glands, Skene's glands, minor vestibular glands, aberrant mammary tissue, or endometriotic implants. They are characterized by a remarkable poorer 5-year survival rate when compared with squamous histotype (32% versus 65% for adenocarcinoma and squamous cell VC, resp.). Intestinal-type adenocarcinoma is a rare variant of vulvar carcinoma with only few cases found in the literature [2–8]. The origin of this neoplasia is still much debated, but the most reliable hypothesis is the origin from cloacal remnants that may persist in the adult in different organs outside the gastrointestinal tract. Similar adenocarcinomas are also found in the uterine cervix and vagina, and the diagnosis is based on the presence of goblet cells or colorectal morphology and “intestinal-type” immunohistochemical phenotype. Because of its extremely low incidence and very few reports available in the literature till date, the optimal management of these patients is still debated.

Hereby, we report the case of a woman affected by advanced intestinal-type vulvar adenocarcinoma, who achieved a complete clinical and pathological response after neoadjuvant chemotherapeutic treatment with platinum and paclitaxel.

## 2. Case Presentation

**Figure 1:**
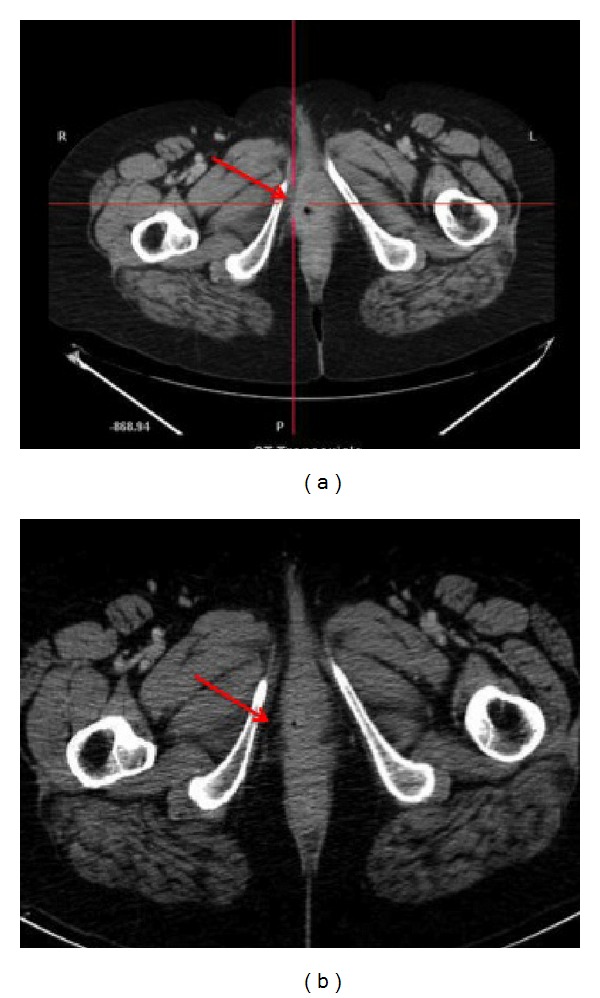
TC images of vulvar cancer lesion before (a) and after (b) neoadjuvant chemotherapy.

**Figure 2:**
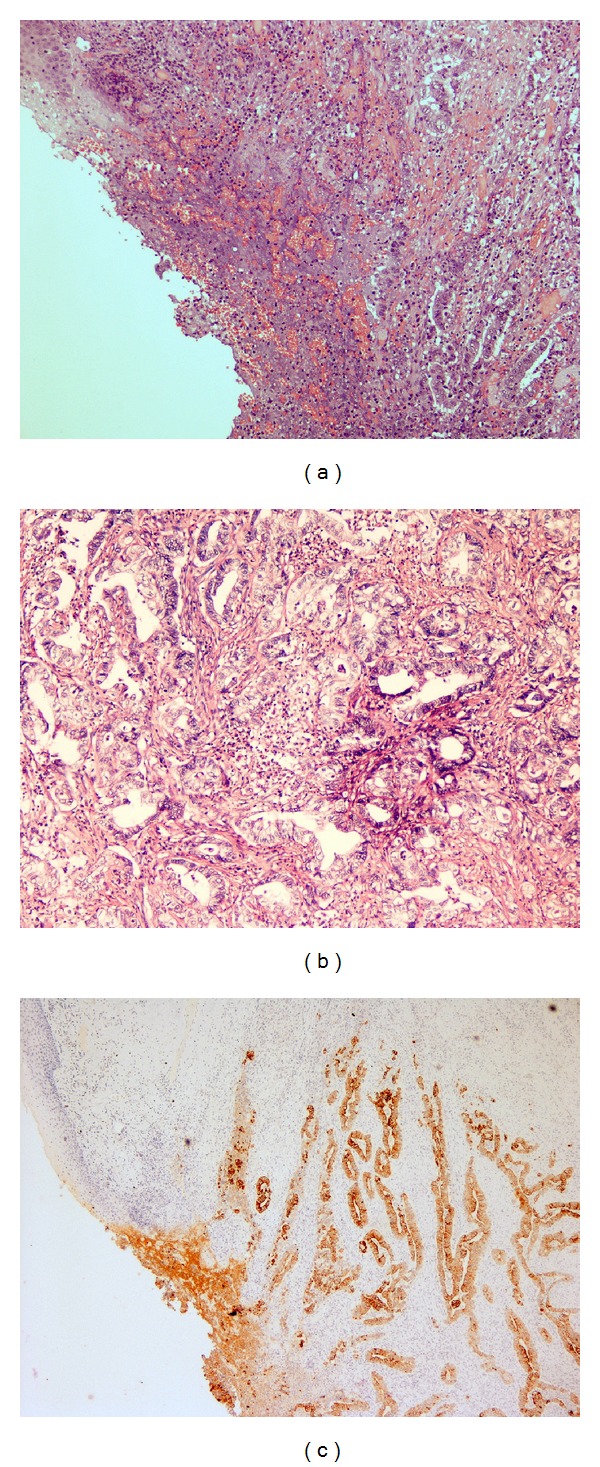
(a) Ulcerated lesion of the uterine cervix: the bottom of the ulcer shows an adenocarcinomatous proliferation. Haematoxylin-eosin, 100x. (b) Higher magnification of the malignancy, showing a moderately differentiated adenocarcinoma (G2). (c) Immunohistochemical expression of cytokeratin 20 in the neoplastic glands.

A 57-year-old woman referred to our institution complaining of a vulvar mass associated with bleeding from external genitals for 3 months. On recovery at our department, she was submitted to an accurate physical examination, which revealed an easily bleeding dome-shaped vulvar lesion on the right major labium measuring 5 cm in diameter, which involved the caudal portion of the vagina and the posterior vulvar fork. Moreover, a palpable lymph node was found in the ipsilateral inguinal area. Blood exams revealed mild anemia but were negative for other abnormalities. The patient was extremely in pain at gynecological examination and she was submitted to rectovaginal examination under narcosis, which confirmed extension of the disease (FIGO stage III). Multiple biopsies of the lesion and nearer tissues were performed with histological finding of a moderately differentiated invasive adenocarcinoma of the vulva ulcerating the surface epithelium. The neoplastic glands showed a colorectal morphology. The immunohistochemical pattern was strongly positive for cytokeratin 20, CEA, CDX2, and p16, with weak and focal positivity for cytokeratin 7, while vimentin and estrogen receptors were negative (Table 1). These findings, along with the microscopical features of the tumor, were supportive for “intestinal-type adenocarcinoma of the vulva” (also known as “adenocarcinoma of cloacogenic origin”). Gastroenteric endoscopy and colonoscopy failed to show suspected lesions. Serum tumoral markers (AFP, CEA, CA125, CA15.3, and CA19.9) were all within normal limits. The abdomen-pelvic computed tomography (CT) revealed an enlarged area in the right vulvovaginal wall (Figure 1(a)) and right inguinal enlarged lymph node, measuring 2 cm in the largest diameter. Because of the advanced stage of disease close to the urethra, anus, and vagina, a neoadjuvant chemotherapic treatment was proposed to the patient. After adequate patient counseling and signing informed consent, three cycles of paclitaxel 175 mg/m^2^ plus cisplatin 100 mg/m^2^ were administrated every 3 weeks. Neither adverse events nor high-grade chemotherapeutic toxicity was reported. On physical examination, performed after the third cycle of chemotherapy, the vulvar lesion was markedly reduced and this finding was confirmed at abdomen-pelvic CT scan, which showed no evidence of the disease (Figure 1(b)), with regression of inguinal lymphadenopathy. The patient underwent surgical excision of the vulvar lesion with en bloc removal of a vaginal segment measuring 4.5 cm in length plus adjacent vulvar skin and right groin dissection. At macroscopic histological examination, a vulvar nodule measuring 1.5 cm in diameter was found; microscopically, this lesion presented pathological and immunohistochemical features of vulvar adenocarcinoma of colorectal type (Figures 2(a)-2(b)). The tumor invaded up to the subcutaneous adipose tissue, with free surgical margins and negative superficial and deep inguinal lymph nodes. No major perioperative complications were recorded. The patient was earlier discharged and she is still alive with no evidence of disease 4 months after the surgery.

## 3. Discussion

“Intestinal-type” vulvar adenocarcinoma is a very uncommon neoplasia and, consequently, its clinical behavior and prognosis are poorly understood and studied. Only few cases of this variant of vulvar adenocarcinoma have been previously described (Table 1) [2–11]. The majority of them arose in the vestibular region and only once showed metastatic lymph node disease [10]. No well-established diagnostic and therapeutic criteria have been proposed, as most of the reported cases focused their attention on the histopathological and immunophenotypical features of the tumor. Most of the cases showed a polypoid macroscopic appearance and villoglandular microscopic features, with occasional or abundant goblet or Paneth cells. As for all the described vulvar intestinal-type adenocarcinomas, our case reports a tumor arising in continuity with the epidermis, but unrelated to the underlying mucous glands. It stained positive for cytokeratin 20 (Figure 2(c)), CDX2, and CEA, as colonic cancers do, but was also immunoreactive for cytokeratin 7 and p16, which are characteristic for the female genital tract neoplasms [12]. We are not aware of other vulvar intestinal cancers expressing CDX2, as this marker was not investigated in the published studies. Nuclear CDX2 immunoreactivity is found in small and large intestinal epithelium. Wherever others will confirm its expression in this subtype of vulvar adenocarcinoma, it would represent a confirmation of the cloacal origin of these neoplasms. Similar to this case, cervical invasive intestinal-type adenocarcinomas show diffuse expression of CDX2, CK20, CEA, and p16. They preserve their CK7 immunoreactivity and are usually p16 positive [13].

Interestingly, the positivity of the present tumor for p16 suggests that vulvar intestinal-type adenocarcinoma could be associated with high-risk HPV. Furthermore, as described earlier, no increased serum tumoral markers (AFP, CEA, CA125, CA15.3, and CA19.9) have been found in our case, consistently with reports previously described in the literature [4–6, 8–10].

The clinical behavior of this rare malignant cancer seems to be rather indolent, with progression-free interval ranging from 12 to 120 months [2–11]. Generally, patients affected by this neoplasia underwent different surgical options like radical vulvectomy with unilateral or bilateral inguinal-femoral lymphadenectomy [13] or unilateral emivulvectomy with radical groin lymph node dissection [14, 15] or wide excision of the lesion in case of very small tumors (<2 cm). Lymph node metastasis was demonstrated in only one case reported by Cormio et al. [10]. Data coming from reports available in the literature suggest a good response to surgical and chemotherapeutic treatments even if most of them will relapse. According to our findings, Cormio et al. reported on an advanced well-differentiated adenocarcinoma of intestinal-type of the vulva with complete response to first-line chemotherapy but with a recurrence-free interval of 6 months, thus suggesting an initial therapeutic role of chemotherapy in these cases [10]. Several authors have recently advocated the role of neoadjuvant chemotherapy followed by radical surgery as a compelling option for vulvar cancer patients, capable of reducing the need for demolitive and disfiguring surgery by decreasing the tumor volume and minimizing perioperative complications with the aim of improving the quality of life of these patients. Rationale for platinum-paclitaxel association is strong and comes from both in vitro and clinical studies. In fact, it has been demonstrated that, despite considerably different mechanisms of action, these two drugs can, when appropriately sequenced, augment the effects of each other; it has also been shown that this association can have a supra-additive growth-inhibitory effect on ovarian carcinoma cell lines and a clear additive or supra-additive cytotoxic effect on the vulvar SCC cell lines [16].

According to our experience and the good response to chemotherapy reported until now for this setting of tumors, we could consider neoadjuvant platinum/paclitaxel chemotherapy followed by surgery as a valuable option for advanced vulvar intestinal-type adenocarcinoma not susceptible to primary surgical treatment. Both pathologists and gynecologic oncologists should be aware of the existence of this rare tumor. However, more cases with longer follow-up period are needed to fully understand its origin and better establish the biomolecular pattern of the disease, to elucidate the best therapeutic management and its long-term prognosis.

## Figures and Tables

**Table 1:** The literature reported vulvar intestinal adenocarcinoma cases.

Author, year	PTS age	Surgical treatment	Adjuvant treatment	Immunohistochemical pattern	PFS (months)
Tiltman and Knutzen, 1978 [5]	50	Modified RV + GD	NR	Not performed	12
Kennedy and Majmudar, 1993 [3]	Case 1: 54 years Case 2: 63 years	RV + bilateral GD Wide LE	NR NR	CK+ CEA−	120 48
Willen et al., 1999 [6]	57	Wide LE	NR	CK17+ Broad CK+ Polyclonal CEA+− Chromogranin A+−	26
Nasir et al., 2001 [4]	43	EV + bilateral GD	NR	CEA+ p53+ Broad CK+ Ras10+− ER− PR− Bcl2− c-erB2−	18
Rodriguez et al., 2001 [7]	69	Wide LE	NR	CK7+ CK20+− OC125 apical CEA apical OC19.9 apical	36
Liu et al., 2003 [9]	49	LE + bilateral GD	NR	Not performed	24
Dubè et al., 2004 [8]	58	Radical EV + ipsilateral GD	NR	CK20+− CK7+ ER− PR−	16
Ghamande et al., 1995 [2]	67	RV + bilateral GD	NR	CEA+	17
Cormio et al., 2012 [10]	Case 1: 58 years Case 2: 42 years	EV + GD RV + GD	Chemotherapy NR	CK7+ CK 20+−	54 39
Karkouche et al., 2012 [11]	31	LE	NR	CK 20+ CK 7−	15
Our case	57	NACT + LE + ipsilateral GR	—	CK 20+ CEA+ P16+ CDX2+ focal Ca125+− ER− CK 7− Vimentin−	17

LE: local excision, E: emivulvectomy, RV: radical vulvectomy, GD: groin dissection, NACT: neoadjuvant chemotherapy, NR: not reported.
